# The Phytomanagement of PFAS-Contaminated Land

**DOI:** 10.3390/ijerph19116817

**Published:** 2022-06-02

**Authors:** Michael W. H. Evangelou, Brett H. Robinson

**Affiliations:** 1 Institute of Terrestrial Ecosystems, ETH Zürich, Universitätstrasse 16, 8092 Zürich, Switzerland; michaelwassilios.evangelou@usys.ethz.ch; 2 Eberhard Recycling AG, Breitloostrasse 7, 8154 Oberglatt, Switzerland; 3 School of Physical and Chemical Sciences, University of Canterbury, Christchurch 8041, New Zealand

**Keywords:** perfluoroalkyl substances, phytoremediation, leaching, food chain, soil contamination

## Abstract

Globally, several hundred thousand hectares of both agricultural and urban land have become contaminated with per- and polyfluoroalkyl substances (PFAS). PFAS compounds are resistant to degradation and are mobile in soil compared to other common contaminants. Many compounds have K_D_ values (matrix/solution concentration quotients) of <10. PFAS compounds endanger the health of humans and ecosystems by leaching into groundwater, exposure via dust, and, to a lesser extent, through plant uptake. This review aims to determine the feasibility of phytomanagement, the use of plants, and the use of soil conditioners to minimize environmental risk whilst also providing an economic return in the management of PFAS-contaminated land. For most sites, PFAS combinations render phytoextraction, the use of plants to remove PFAS from soil, inviable. In contrast, low Bioaccumulation Coefficients (BAC; plant and soil concentration quotients) timber species or native vegetation may be usefully employed for phytomanagement to limit human/food chain exposure to PFAS. Even with a low BAC, PFAS uptake by crop plants may still exceed food safety standards, and therefore, edible crop plants should be avoided. Despite this limitation, phytomanagement may be the only economically viable option to manage most of this land. Plant species and soil amendments should be chosen with the goal of reducing water flux through the soil, as well as increasing the hydrophobic components in soil that may bind the C-F-dominated tails of PFAS compounds. Soil conditioners such as biochar, with significant hydrophobic components, may mitigate the leaching of PFAS into receiving waters. Future work should focus on the interactions of PFAS with soil microbiota; secondary metabolites such as glomalin may immobilize PFAS in soil.

## 1. Introduction

### 1.1. Sources of PFAS Compounds and Their Potential Effects on Human Health

Per- and polyfluoroalkyl substances (PFAS) comprise >4000 anthropogenic compounds [1], characterized by numerous stable C-F bonds that render PFAS compounds resistant to degradation in the environment [2]. PFASs have been used since the mid-20th century, with production peaking in 2000–2002 at 4650 t yr^−1^ when their use was discouraged due to concerns about environmental persistence and human toxicity [3,4,5]. Human exposure to PFAS has been linked to high cholesterol [6], thyroid disease [7], delayed child development and poor maternal health [8], ulcerative colitis [9], as well as kidney and testicular cancer [10].

PFASs are used as surfactants, for example, perfluorooctane sulfonic acid (PFOS), and in products containing non-stick, stain, and water-resistant coatings such as packaging, clothing, carpets, outdoor textiles and sporting equipment, ski and snowboard waxes, non-stick cookware, cleaning agents and fabric softeners, polishes and waxes, and latex paints, pesticides and herbicides, hydraulic fluids, windshield wipers, paints, varnishes, dyes, and inks, adhesives, medical products, and personal care products [4,11,12,13,14]. The manufacture, use, and disposal of PFAS-containing products have resulted in pervasive environmental contamination, including many thousands of hectares of soil [15,16,17].

### 1.2. Phytomanagement and Review Objectives

There are several reports that plants could be used to improve PFAS-contaminated soils via phytoremediation. These approaches include phytoextraction [18,19], which would require repeated cropping and appropriate disposal of PFAS-rich biomass, phytostabilisation, i.e., immobilization in the soil [20], or degradation in the root zone [20,21]. Phytoremediation requires that the area be left in vegetation until either the contaminant has been removed (phytoextraction) or degraded to the point where it no longer poses a risk to humans and ecosystems [22,23]. If the goal is simply to eliminate the movement of PFAS, then the area needs to be left undisturbed in perpetuity [24]. Depending on the value of the land and the timescales involved, phytoremediation may be prohibitively expensive [22], even if the annual costs are lower than alternative technologies such as soil washing or soil removal [25]. The costs of phytoremediation may be reduced, or even eliminated if valuable biomass can be grown on the site, while contemporaneously mitigating the risks posed by PFAS. Such “phytomanagement” [26] may render the cleanup time unimportant.

This review aims to determine the feasibility and critical success factors of phytomanagement for PFAS-contaminated soils. Specifically, we seek to determine (i), the extent of PFAS-contaminated soils where phytomanagement could be applied, (ii) the mobility of PFAS in the soil-plant system, (iii) how vegetation management may affect the risks posed by PFAS-contaminated soils, and (iv) critical knowledge gaps required to increase the success of phytomanagement.

## 2. Chemical Properties of PFAS Compounds Affecting Phytomanagement

The PFAS are divided into two primary categories: polymer (e.g., polytetrafluoroethylene (PTFE), commonly known by the brand name Teflon) and non-polymer moieties, which are the most pernicious environmental contaminants. The family of non-polymer PFAS encompasses two major classes: perfluoroalkyl substances and polyfluoroalkyl substances. Perfluoroalkyl substances are fully fluorinated (perfluoro-) alkane (carbon-chain) molecules. The most encountered perfluoroalkyl substances are perfluoroalkyl acids (PFAAs), which include perfluoroalkyl carboxylic acids (PFCAs), such as perfluorooctanoic acid (PFOA), and perfluoroalkane sulfonic acids (PFSAs), such as PFOS. Polyfluoroalkyl substances are distinguished from perfluoroalkyl substances by not being fully fluorinated. Of the polyfluoroalkyl substances most common are fluorotelomer substances, which include fluorotelomer alcohols (FTOHs), fluorotelomer sulfonic acids (FTSAs), fluorotelomer carboxylic acids (FTCAs) [27].

Certain key properties of fluorine (F) profoundly influence the chemical and physical properties of PFAS. The high electronegativity and small size of fluorine lead to a strong C-F bond, which results in the thermal and chemical stability of PFAS. PFCAs and PFSAs can decompose at >300 °C but complete mineralization occurs at temperatures >1000 °C [28]. During thermal degradation of PFAS, gaseous PFAS (for example CF_4_, C_2_F_6_ or •CF_3_, •C_2_F_3_) can be formed during [28], which are potent greenhouse gases. The high electronegativity of fluorine also leads to polar bonds with a partial negative charge towards F, resulting in the case of acidic functional groups (such as carboxylic or sulfonic acid) to low pKa [29]. Specific pKa values for PFAAs are generally not available, but Vierke et al. [30] estimated that the pKa values of perfluorobutanoic acid (PFBA), perfluorohexanoic acid (PFHxA), perfluoroheptanoic acid (PFHpA), PFOA, perfluorononanoic acid (PFNA), perfluorodecanoic acid (PFDA), and perfluoroundecanoic acid (PFUnA) are <1.6. Under ambient environmental conditions, PFAAs and PFSAs can be found in a dissociated state, thus the water solubility is rather high (e.g., PFBA 560 g L^−1^, PFHxA 21.7 g L^−1^). In the case of fluorotelomers hydrophobic properties prevail, thus the water solubility is low (e.g., 4:2 FTOH, 0.07 g L^−1^). Generally, the water solubility decreases with an increasing number of perfluorinated C-atoms. The volatility of the PFAS varies strongly depending on the PFAS themselves and the environmental conditions. For PFAAs and PFSAs the transition into the gas phase is low and depends largely on soil pH. For example, the protonated form of PFOA is moderately volatile, while the volatility of the dissociated PFOA is negligible [31]. Fluorotelomers, on the other hand, are highly volatile with FTOH exhibiting higher volatility than the FTS [32].

## 3. Potential Areas Where PFAS Phytomanagement Could Be Deployed

### 3.1. Low-Value Lands

Phytomanagement is likely to be the most economically viable land management option for low-value land [26,33], where there is little economic incentive to re-develop the land following the deployment of high-cost alternative remediation techniques such as soil washing or soil removal. Areas suitable for phytomanagement include small areas of low-value urban land [34], often lands associated with industrial activity, and large areas of (former) agricultural land [24].

Lower value sites in industrial areas may arise from point sources of PFAS contamination in the soil, including sites where Cr(VI) electroplating or Aqueous Film-Forming Foam (AFFF) have been used. These account for some 80% of contaminated sites, although a much smaller proportion of the contaminated area [35,36,37]. PFAS has been used for both electroplating and as a component of AFFF [38], which is used to fight fires or reduce the risk of fire. AFFF is used in chemical plants, flammable liquid storage and processing facilities, merchant operations (oil tankers, offshore platforms) municipal services (fire departments, firefighting training centers), oil refineries, terminals, and bulk fuel storage farms, aviation operations (aircraft rescue and firefighting, hangars) and military facilities [39,40].

Larger areas that may be suitable for phytomanagement include soils affected by landfill leachate and the application of PFAS-contaminated biosolids, compost, or manure. In Antwerp (Belgium), the air emissions from a 3 M fluorochemical plant have contaminated the soil up to a distance of 3 km from the plant, with concentrations reaching up to 202 µg kg^−1^ PFOS in the vicinity of the fluorochemical plant; up to 21 µg kg^−1^ at a distance of 2.3 km and up to 4.5 µg kg^−1^ at a distance of 3 km from the plant (Groffen et al., 2019). PFAS emissions from fluorochemical plants can be transported in the atmosphere over hundreds of kilometers either in the gas phase, as nonionic precursor compounds such as FTOHs, or by sorption to airborne particles, such as ionic perfluoroalkyl acids (PFAAs), thus reaching remote regions in North America, Asia, and Europe [41,42].

Between 2000 and 2006, farmers spread 27,700 tons of biosolid, compost, and manure on ca. 800 agricultural sites in the catchment areas of the rivers Moehne and Upper-Ruhr. Overall, 53,000 tons of the mixture were applied to >1300 areas in North Rhine-Westphalia [15]. Several hectares showed PFOS and perfluorooctanoic acid (PFOA) concentrations up to 6300 μg kg^−1^ Brilon-Scharfenberg [16] and up to 30,000 μg kg^−1^ PFAS in Soest [35]. The application of the PFAS-contaminated conditioner caused the contamination of drinking water (500–640 ng L^−1^ PFOA), resulting in 4.5–8.3 times higher PFOA levels in the blood plasma of affected people than those for the reference population [16]. Another prominent PFAS soil contamination case, caused by the application of additives to the soil can be found in Rastatt, Baden-Württemberg, Germany. As of August 2018, 644 hectares of soil in Landkreis Rastatt and Stadtkreis Baden-Baden, as well as 240 hectares in Mannheim, are expected to be contaminated by PFAS. The most likely cause of this PFAS contamination is the use of compost blended with contaminated paper mill waste, which was applied to agricultural land between 2005 and 2008 [17].

### 3.2. Phytomanagement of Areas Affected by PFAS from Landfill Leachate

Landfill leachate will be a major source of PFAS for decades due to the leaching of PFAS into surface and groundwater, which may then be used for irrigation [43]. PFAS contaminants originating from three sites used by the 3 M Corporation for disposal of PFAS manufacturing wastes over several decades have resulted in an East Metro PFAS groundwater contamination plume currently covering over 150 square miles, affecting the drinking water supplies of over 140,000 Minnesotans [44].

Table 1 shows that landfill leachate concentrations typically exceed 1000 ng L^−1^. However, these reports probably underestimate the total emission of PFAS from municipal solid waste landfills as significant amounts of unknown precursors and short-chain (C2-C3) PFAS can be released into the environment through leachates and the process of volatilization [45]. Fuertes et al. [46] and Yan et al. [43] reported an increase in PFAS concentrations in landfill leachates after biological treatments, such as an external membrane bioreactor. This increase could be explained by the possible degradation of PFASs precursors such as fluorotelomer alcohols or fluorotelomer sulfonates [46].

## 4. PFAS Fluxes in the Soil-Plant System

### 4.1. PFAS Solubility and Mobility in Soil

The immobilization and/or degradation of PFAS in contaminated soil depends on the chemical properties of the individual PFAS compounds. The defining characteristic of PFAS is a stable C-F bond, which renders PFAS compounds resistant to both chemical and biological degradation [52]. Saturated polytetrafluoroethylene (PTFE) is entirely inert below 400 °C [53]. While PTFE is insoluble in water and relatively immobile in the soil-plant system, PFOA and PFOS are relatively mobile, with KD values typically <10 [54,55].

Owing to the molecular structure of PFAAs, which have a hydrophobic and lipophilic carbon-fluorine “tail” and a polar and hydrophilic non-fluorinated “head”, the portioning mechanisms affecting PFAAs include hydrophobic and lipophobic effects, electrostatic interactions, and interfacial behavior [4]. Thus, the sorption of PFAAs cannot be predicted by a single property of the soil or a single property of the PFAAs (i.e., sorbate). Nonetheless, soil organic carbon is the most important factor in PFAS immobilization in soil [56].

There is a positive linear relation between PFAS sorption and soil organic carbon content [57,58], as well as between sorption and length of the hydrophobic carbon-fluorine “tail” [59]. Compared to CF_2_, any CH_2_ groups present play a subordinate role, as the example of 6:2 FTS (fluorotelomer sulfonate) shows, which sorbs 40% less to soil than PFOS, although both have the same number of C-atoms and the same sulfonic acid functional group. The sorption of 6:2 FTS equals more than that of PFHxS, which has the same number of perfluorinated C-atoms [60]. As a consequence, short-chain PFSAs (e.g., PFBS, perfluorobutane sulfonic acid, and short-chain PFCAs (e.g., PFHxA) are retarded less than their long-chain counterparts and are thus more mobile. Owing to the high mobility of short-chain PFAAs, PFAAs create generally longer plumes than those of total petroleum hydrocarbons, polycyclic aromatic hydrocarbons, methyl tert-butyl ether, and even chlorinated hydrocarbons [61]. In addition, PFSAs tend to sorb more strongly than PFCAs of equal chain length [57], probably because the sulfonic acid group is larger than the carboxylic group, thus displaying a lower charge density and a slightly higher hydrophobicity [62].

The sorption of PFAAs to soil is influenced by pH and ionic strength [20]. Sorption of PFAS decreases with increasing pH. This effect is not due to deprotonation, because PFAAs are deprotonated at environmentally relevant pHs [63]. Instead, pH-dependent changes in the soil organic carbon surface charge are the cause of the pH-dependent sorption of PFAS [57]. At ambient environmental conditions (pH > 5), solutions with high ionic strength tend to promote the sorption of PFAS onto mineral surfaces by probably suppressing the electrostatic repulsive force [64]. Electrostatic interactions of PFAS with clay minerals and iron oxides are only important when the organic carbon content is low [65].

Maintenance of soil pH in the lower range that is optimal for plant growth (pH 5–6) is more likely to reduce PFAS mobility. Given that trace element cations are more mobile under acidic conditions, a suitable pH must be selected where the mobility of all contaminants is kept within acceptable levels.

Polar PFAS compounds in soil solution can affect the soil biota. In former fire-training areas where PFOS has been used, soil concentrations of 3.4–532 µg kg^−1^ resulted in distinct bacterial communities, with concentrations >100 µg kg^−1^ significantly reducing biodiversity [66]. The presence of 0.1–100 µg kg^−1^ sodium p-perfluorous nonenoxybenzene sulfonate increased the archaea:bacteria ratio in grassland soils, which will likely the rate affect ammonia oxidation [67]. Endophytic microorganisms can significantly reduce the toxicity of PFAS compounds to plant roots [68].

### 4.2. Plant Uptake of PFAS Compounds

Plant uptake of PFAS in phytomanagement risks increasing the likelihood of PFAS entering the (human) food chain. Species selected for plant uptake experiments have largely been crop species. However, some species, including *Salix* spp., *Equisetum* sp. and *Brassica juncea* have been tested for their potential to extract PFAS from contaminated soils. Plant uptake can be described in terms of bioaccumulation coefficient (BAC, plant/soil concentration quotient) and translocation coefficient (TC, shoot/root concentration quotient). Much data on the plant uptake of contaminants, including PFAS, is obtained in non-soil environments either directly in a nutrient solution or in an inert medium such as quartz sand that is irrigated with nutrient solution (Table 2). Such experiments remove the complicating interactions with soil, however, they may result in inordinately high BACs due to changes in the roots that increase the apoplastic transfer of contaminants into the stele whence they are translocated to the shoots [69,70]. Table 2 shows some BAC values in non-soil conditions that are manifold higher than in pot soil experiments (Table 3) or field trials (Table 4). Non-soil experiments effectively demonstrate that BACs can vary over an order of magnitude depending on the plant species and the type of PFAS (Table 2), with PFPeA uptake some tenfold higher than PFOS, PFOA, and PFBS. As with other phloem-immobile moieties, PFAS concentrations are higher in the leaves than in the stems.

In hydroponic experiments with *Zea mays*, Krippner et al. [71] reported that BAC decreased at increasing pH, i.e., at pH 5, the uptake of PFDA was significantly higher than at pH 7. They showed that the BACs and TCs short-chain PFASs are higher than long-chain PFAS. Felizeter [80] proposed two mechanisms for the uptake of PFAAs in lettuce: (1) sorption to the tissue between the root surface and the Casparian strip and (2) uptake across the Casparian strip and translocate within plants via vascular tissue. Zhang et al. [81] reported that the sorption to plant tissues between the root surface and the Casparian strip is the dominant process for the uptake of long-chain PFAAs (e.g., PFOS) in *Juncus effusus*, while uptake across the Casparian strip into the vascular tissue might be considered the most relevant mechanism for short-chain PFAAs. Krippner et al. (2014) showed that short-chain PFAAs generally had a TC > 1, thus transferred predominantly and at higher concentrations to the shoots, while long-chain PFAAs displayed a TC < 1, thus being mainly retained in the roots. Similar findings were published by [74] for willow (*Salix eleagnos* L., *S. purpurea* L. and *S. triandra* L.), by [82] for *Bromus diandrus*, by Zhao et al. (2016) for wheat (*Triticum aestivum* L.), by [72] for *Juncus effusus* and by [83] for lettuce (*Lactuca sativa*), which showed that shorter-chain PFAAs were more readily transferred from roots to shoots compared to longer chain PFAAs. The translocation from roots to shoots occurs primarily in the xylem of plants and transpiration is the driving force for nutrient translocation in the xylem. Short-chain PFAAs display a high solubility in water and weak sorption to organic materials [57], thereby facilitating their translocation via transpiration stream to the leafy parts where they accumulate. In contrast, long-chain PFAS, with relatively higher hydrophobicity and lipophilicity tend to be more effectively retained in the roots by the Casparian strip, due to greater interactions with biological macromolecules (e.g., protein, lipid), resulting in their limited upward translocation during transpiration process [41,78,81,83,84].

## 5. Phytomanagement of PFAS-Contaminated Soils

**Figure 1:**
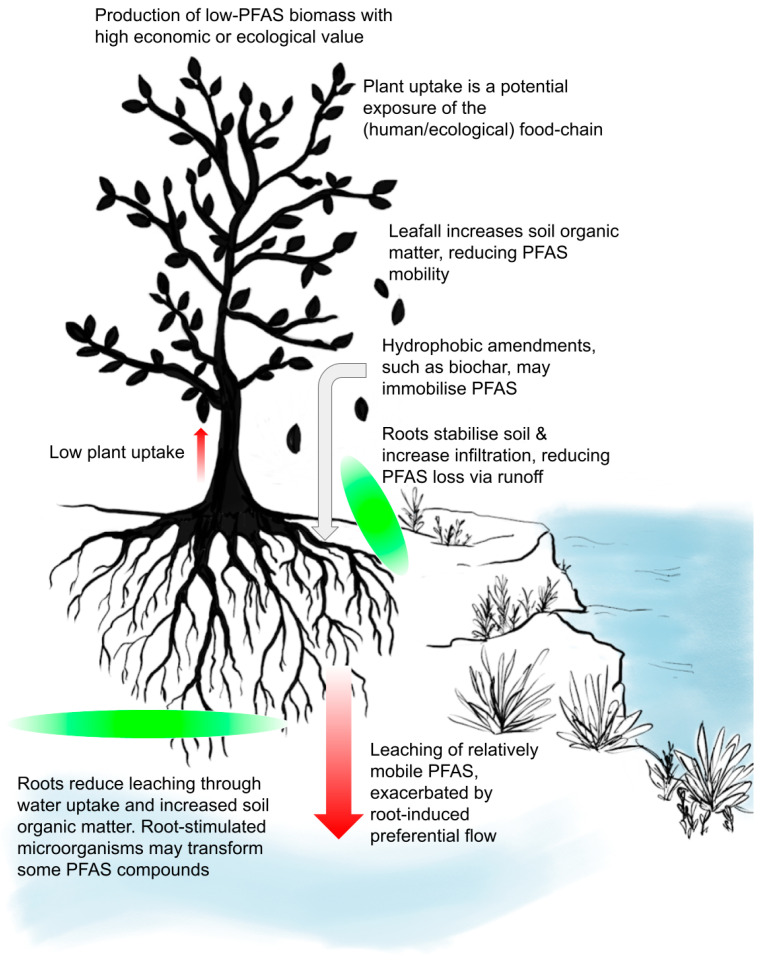
Critical PFAS fluxes in the soil-plant system as affected by plants.

PFAS phytomanagement should mitigate any human or ecological risk of the PFAS in the soil while returning a profit from the land. The selection of appropriate plant species and soil conditioners is critical in achieving this aim because some plants may exacerbate PFAS-mobility (Figure 1). PFAS-contaminated soils pose a potential risk to humans and ecosystems through plant uptake and the entry of PFAS compounds into the (human) food chain. A key first step in PFAS phytomanagement is the identification of:

- The PFAS compounds present and their concentrations.
- The geographical extent of the PFAS contamination.
- The concentrations of any co-contaminants that may be present, especially trace elements such as Cd, which may become mobile in the soil-plant system during phytomanagement.
- The likely exposure pathways through which PFAS compounds may affect humans or ecosystems.

As with other contaminants, direct consumption of soil, either intentionally (pica children) or unintentionally (dust inhalation or dust on consumed food) can result in increased PFAS uptake in humans [85]. Successful phytomanagement should mitigate the leaching of PFAS into receiving waters. Soil, including agricultural land, can be a source of PFAS contamination in waterways, which may then be used as drinking water or for irrigation. Contaminated agricultural land is the most important source of elevated PFAS concentrations in the Ruhr River, which is a tributary of the Rhine [86]. In the United States, up to 80 million people consume drinking water with >10 ng L^−1^ PFOA and PFOS [87].

The success of phytomanagement will depend, to some extent, on the types of PFAS compounds present. The few examples of soils contaminated with PFAS compounds with a low-mobility and toxicity, for example, PTFE, may permit a wide range of species to be used to manage the site. However, in most PFAS-contaminated sites where the compounds present have high mobility and are resistant to degradation, phytomanagement should aim to limit PFAS mobility through hydraulic control and maximization of soil carbon levels. The roots stabilize soil, reducing erosion [88] and decreasing runoff by increasing infiltration. Soil amendments including composts, lignite coal [89], or biochar [90], may be particularly effective because they contain hydrophobic components [91] that may immobilize PFAS by binding to the CF-dominated nonpolar tail. Plant species, such as grasses, that promote the accumulation of soil carbon may be preferred over trees, which may result in depletion of soil carbon, even if greater evapotranspiration from trees may reduce water flux through the soil [92]. Plowing and the use of mineral fertilizers should be minimized as these practices reduce soil carbon [93] and may increase dust, which may transport PFAS-compounds offsite.

### 5.1. Use of Crop Plants in Phytomanagement

If food or fodder crops are to be used as part of a phytomanagement solution for contaminated land, then the products they generate must not contain unacceptable PFAS concentrations. The European Food Safety Authority has opined that the Tolerable Weekly Intake (TWI) for PFOS and PFOA should be 13 ng kg^−1^ b.w. and 6 ng kg^−1^ b.w. respectively [94]. This value may be exceeded if crops are irrigated with water that meets the current USEPA’s health advisory guideline of 70 ng L^−1^ [95]. Plant uptake experiments in greenhouse and field conditions (Table 3 and Table 4, respectively) indicate that when grown in PFAS-contaminated soil, plant uptake of PFOS and PFOA can exceed >1000 µg kg^−1^. This indicates that consumption of just a few grams of this material would be sufficient to exceed the TWI. Given BACs in the range of 0.1–3, and weekly consumption of 250 g (d.w.) of a crop, then the threshold soil concentrations for food safety would be 1.2–36.4 µg kg^−1^ for PFOS and 0.56–16.8 µg kg^−1^ for PFOA. Most of the soils described in Section 3.1 exceed these concentrations, indicating that if phytomanagement is to generate a profit from PFAS-contaminated land, it cannot occur using crop plants that would be consumed by humans. Instead, phytomanagement must employ plant species that generate valuable products such as timber, that are either outside the human food chain or have produced products such as honey or essential oils that are consumed in insufficient quantities to pose a human health risk. Such products would need to be extensively tested to ensure PFAS concentrations did not pose a human health risk. While there is a growing body of literature on PFAS in agricultural plants [96], there is a lacuna of information on the BACs for plants that may be used for ecological restoration of PFAS-contaminated soils, for example in the construction of green corridors or green spaces in urban areas.

### 5.2. PFAS Phytoextraction Is Likely Infeasible

Potentially, successive crops of plants that take up high concentrations of PFAS could be used to extract PFAS-compounds from contaminated soil, eventually leading to soils with PFAS-concentrations within acceptable limits. For example, growing crops of *Salix viminalis* (assuming a BAC of 3.0 and a removable biomass production of 10 t ha^−1^ yr^−1^) on soil contaminated with 1000 µg kg^−1^ PFOA (down to 0.15 m) would require 65 years (assuming annual coppicing) to halve the soil PFOS concentration, which would still be >20 fold higher than concentrations shown to result in unacceptable concentrations in food crops (Section 3.1). Remediations are increased by contaminant heterogeneity as well as co-contamination [22]. If phytoextraction can be combined with the generation of e.g., bioenergy, then these cleanup times may be unimportant [33]. One advantage that PFAS phytoextraction may have over the phytoextraction of trace elements is that incineration of the PFAS-containing biomass may destroy the PFAS, hence reducing the disposal costs of the resultant ash. Nevertheless, the long-term intensive management required for phytoextraction is likely to make this option more expensive than other remediation methods. Therefore, similar trace element phytoextraction, PFAS phytoextraction may find little commercial application [22] unless the BACs for PFAS compounds can be increased by an order of magnitude.

## 6. Conclusions

Given the recalcitrance of most PFAS compounds, it is unlikely that phytomanagement will result in site clean-up, either through in situ degradation or by phytoextraction, in a timeframe that is relevant to the economic cycle (<100 years). The presence of co-contaminants, such as trace elements, will only increase clean-up times. Therefore, phytomanagement should focus on minimizing the mobility of PFAS compounds while maximizing profits from the land. Crop plants with low BACs may still exceed current Food Safety Standards and should therefore not be used. There is insufficient information to determine whether crop plants could be safely used as animal fodder, however, negative public perception may limit the viability of food produced on contaminated land, and this perception may detrimentally affect food profitability in the entire region. In contrast, low BAC non-food crops, timber species, or native vegetation may be usefully employed for phytomanagement to limit human and food chain exposure to PFAS. Future work should identify plant species and soil amendments that reduce water flux through soil, as well as increase the hydrophobic components in soil that may bind the C-F-dominated tails of PFAS compounds. Soil conditioners such as biochar, with significant hydrophobic components, may mitigate the leaching of PFAS into receiving waters. A critical research gap is the interactions of PFAS with soil microbiota and whether secondary metabolites such as glomalin may immobilize PFAS in soil.

## Figures and Tables

**Table 1:** PFAS concentrations in Landfill leachates.

Location	Landfill Leachate PFAS Concentration (ng L^−1^)	Reference
Spain	1082	[46]
Finland	403	[47]
Norway	4157	[48]
Sweden	26,454	[49]
Australia	1365 and 5254	[50]
USA	2253–6157	[51]
China	Up to 292,000	[43]

**Table 2:** Response of plants to PFAS compounds * in non-soil experiments.

Plant Species	PFAS	Soln. Conc. (µg L^−1^)	Plant Conc. (µg kg^−1^)	BAC *
[71]				
*Zea mays* L.	7 PFCAs 3 PFSAs	each 100	Root 0.12–3.63	
[72]				
*Juncus effusus* L	4 PFCAs 3 PFSAs	4635	Root < 150,000 Shoot < 4000	<1 (all compounds)
[73]				
*Triticum aestivum* L.	PFBA PFHpA PFOA PFDoA	each 1000	Root 200 PFBA 550 PFHpA 600 PFOA 10,000 PFDoA Shoot 450 PFBA 200 PFHpA 150 PFOA 100 PFDoA	n.r. n.r. n.r. n.r. 0.85 PFBA 0.46 PFHpA 0.43 PFOA 0.33 PFDoA
[74]				
*Salix* sp.	9 PFCAs 2 PFSAs	each 10	Root 10–300 Shoot 5–315	0.5 (PFDA)-31.5 (PFBA)
[18]				
*Festuca rubra* L.	PFHxS PFOS PFOA PFPeA PFHxA PFBS	890 850 940 1600 2100 920 Solution irrigated onto sand columns	1146 (PFOS)-21,882 (PFPeA)	11 (PFOS)-111 (PFPeA)
*Cynodon dactylon* (L.) Pers.			220 (PFOS)-4642 (PFPeA)	2.0 (PFOS)-22 (PFPeA)
*Schedonorus arundinaceus* Schreb.			264 (PFOS)-14,780 (PFPeA)	1.4 (PFBS)-71 (PFPeA)
*Helianthus annuus* L.			78 (PFOS)-3937 (PFPeA)	0.7 (PFOS)-18 (PFPeA)
*Brassica juncea* L.			424 (PFOS)-13,030 (PFPeA)	3.7 (PFOS)-60 (PFPeA)
*Amaranthus tricolor* L.			326 (PFBS)-38,121 (PFPeA)	2.6 (PFBS)-176 (PFPeA)
*Equisetum hyemale* L.			40 (PFBS)-32,032 (PFPeA)	0.3 (PFBS)-147 (PFPeA)
*Salix nigra* Marshall			Leaf 556 (PFOS)-31,646 (PFPeA) Stem 11 (PFOS)-373 (PFPeA)	5.2 (PFOS)-156 (PFPeA) n.r.
*Fraxinus pennsylvanica* Marshall			Leaf 1 (PFOS, PFOA)-169 (PFPeA) Stem 21 (PFOS)-379 (PFPeA)	<0.3 (all compounds) n.r.
*Pinus taeda* L.			Leaf 13 (PFOS)-964 (PFPeA) Stem 1 (PFBS)-3 (PFPeA)	0.1 (PFOS)-4.9 (PFPeA) n.r.
*Betula nigra* L.			Leaf 1759 (PFOS)-28,496 (PFPeA)	9.8 (PFBS)-142 (PFPeA)
*Liquidambar styraciflua* L.			Leaf 392 (PFOS)-2070 (PFPeA) Stem 7 (PFAS)-981 (PFPeA)	2.6 (PFBS)-11 (PFOA) n.r.
*Platanus occidentalis* L.			Leaf 262 (PFOS)-17,838 (PFPeA) Stem 2 (PFAS)-83 (PFPeA)	2.5 (PFOS)-90 (PFPeA) n.r.
*Liriodendron tulipifera* L.			Leaf 814 (PFOS)-35,975 (PFPeA) Stem 1 (PFOS, PFAS, PFBS)-1276 (PFPeA)	n.r.

* Bioaccumulation coefficient (BAC), Perfluorobutanoic acid (PFBA), Perfluorobutane sulfonic acid (PFBS), Perfluorodecanoic acid (PFDA), Perfluorododecanoic acid (PFDoA), Perfluoroheptanoic acid (PFHpA), Perfluorohexanoic acid (PFHxA), Perfluorohexane sulfonic acid (PFHxS), Perfluorononanoic acid (PFNA), Perfluorooctanoic acid (PFOA), Perfluorooctane sulfonic acid (PFOS), Perfluoropentanoic acid (PFPeA), Perfluoroundecanoic acid (PFUnA). n.r. = not reported.

**Table 3:** Response of plants to PFAS compounds * in pot experiments.

Plant Species	PFAS	Treatment and Soil Conc. (µg kg^−1^)	Plant Conc. (µg kg^−1^)	BAC *
[75]				
*Daucus carota* L.	PFOS, PFOA	Spiked soil + compost PFOA 500 PFOS 500	Peel 250 PFOA 175 PFOS Core 146 PFOA 201 PFOS Leaves 999 PFOA 664 PFOS	0.2–0.61 PFOA 0.10–0.58 PFOS 0.05–0.36 PFOA 0.10–0.64 PFOS 0.80–3.43 PFOS 0.62–2.26 PFOS
*Lactuca sativa* L.	PFOS, PFOA	Spiked soil + compost PFOA 500	Heart 2540 PFOA 1680 PFOS Leaves 1030 PFOA 77 PFOS	4.19–4.87 PFOA 2.94–3.49 PFOS 1.59–2.13 PFOA 0.11–0.19 PFOS
[76]				
*Zea mays* L.	PFOS, PFOA	Spiked soil each 1000	Straw: 126 PFOA 104 PFOS Corn: 4 PFOA 3 PFOS	0.126 PFOA 0.104 PFOS 0.004 PFOA 0.003 PFOS
*Triticum sativum* L.	PFOS, PDOA	each 1000	Straw: 1900 PFOA 270 PFOS Corn: 9 PFOA <0.1 PFOS	1.9 PFOA 0.27 PFOS 0.009 PFOA <0.1 PFOS
[77]				
*Zea mays* L.	7 PFCAs 3 PFSAs	Spiked soil 250 and 1000 each	Straw 500 (PFDA)-35,000 (PFBA) Kernel <LOQ-380 (PFPeA)	0.04 (PFDA)-35.2 (PFBA) <LOQ-0.38 (PFPeA)

* Bioaccumulation coefficient (BAC), Perfluoroalkyl carboxylic acids (PFCAs), Perfluorooctanoic acid (PFOA), Perfluorooctane sulfonic acid (PFOS), Perfluoroalkyl sulfonates (PFSAs), Perfluoropentanoic acid (PFPeA).

**Table 4:** Response of plants to PFAS compounds * in field experiments.

Plant Species	PFAS	Treatment and Soil Conc. (µg kg^−^^1^)	Plant Conc. (µg kg^−^^1^)	BAC *
[78]				
*Triticum aestivum* L.	Nine PFCAs Three PFSAs	Biosolids amended soils PFSAs 41.4–220	Root 140–472 (ΣPFAAs) Straws 36.2–178 (ΣPFAAs) Husks 6.15–37.8 (ΣPFAAs) Grains 7.32–35.6 (ΣPFAAs)	1.19–5.18 (ΣPFAAs) 0.24–2.56 (ΣPFAAs) 0.05–0.86 (ΣPFAAs) 0.06–0.92 (ΣPFAAs)
[79]				
*Zea mays* L.	PFOA, PFOS	Spiked soil PFOA ca. 180 PFOS ca. 3000	Shoot: 6.4–7.4 PFOA Shoot: 94–307 PFOS	0.022–0.024 PFOA 0.028–0.063 PFOS
*Lolium perenne* L.	PFOA, PFOS	Spiked soil PFOA ca. 380 PFOS ca. 3150	Shoot: 254–304 PFOA Shoot: 435–1195 PFOS	0.548-0.872 PFOA 0.130-0.255 PFOS

* Bioaccumulation coefficient (BAC), Perfluoroalkyl carboxylic acids (PFCAs), Perfluorooctanoic acid (PFOA), Perfluorooctane sulfonic acid (PFOS), Perfluoroalkyl sulfonates (PFSAs). Sum of total perfluoroalkyl acids (ΣPFAAs).

## Data Availability

Not applicable.
